# Changes in resting-state functional connectivity linked to affective symptoms: insights from a population-based study of adolescents and young adults

**DOI:** 10.1038/s41398-026-04269-y

**Published:** 2026-07-15

**Authors:** Paula M. Henneberg, Katja Beesdo-Baum, Michael Marxen, Garvit Joshi, Kevin Hilbert, Markus Muehlhan, Fabian Huth, Pavol Mikolas, Eva Mennigen

**Affiliations:** 1 Department of Psychiatry and Psychotherapy, TUD Dresden University of Technology, Dresden, Germany; 2 Behavioral Epidemiology, Institute of Clinical Psychology and Psychotherapy, TUD Dresden University of Technology, Dresden, Germany; 3 Department of Psychology, HMU Health and Medical University Erfurt, Erfurt, Germany; 4 Department of Psychology, Faculty of Human Sciences, MSH Medical School Hamburg, Hamburg, Germany; 5 ICAN Institute of Cognitive and Affective Neuroscience, MSH Medical School Hamburg, Hamburg, Germany

**Keywords:** Neuroscience, Biomarkers, Depression, Bipolar disorder

## Abstract

First episodes of affective disorders often emerge during adolescence and young adulthood. Alterations in resting-state functional connectivity (RSFC) have been reported in affective disorders, yet findings are heterogeneous and associations of RSFC with subclinical affective symptoms in community samples remain limited. A better understanding of these underlying neurobiological mechanisms may aid in identifying early vulnerability markers of affective disorders. We examined associations between affective symptom severity and both static and dynamic RSFC using resting-state fMRI data from 512 adolescents and young adults (aged 14–23) drawn from an age- and sex-stratified population-based sample. Group independent component analysis was used to derive RSFC measures. Associations with depressive and manic symptom severity were assessed while controlling for age and sex. Dynamic RSFC was analyzed using a sliding-window approach. Static RSFC showed significant effects of age and sex but no associations with affective symptoms. Dynamic RSFC analysis identified four connectivity states. In one state, manic symptom severity and its interaction with depressive symptom severity were associated with connectivity between the postcentral gyrus and the right superior temporal gyrus. Additionally, the dynamic index fraction of time showed interactions of affective symptoms with age and sex. Overall, RSFC measures demonstrated limited sensitivity to subclinical affective symptom variation in community youth, with only a single state-specific association observed. These findings suggest that subtle alterations in somatomotor-default mode network connectivity may reflect early vulnerability-related processes, though replications and further research are required. Key limitations include the use of very brief symptom measures and developmental heterogeneity across the sample.

## Introduction

Affective disorders, including depression and bipolar disorder, are major global causes of disability and morbidity [1]. They are highly prevalent, frequently comorbid with other psychiatric and medical conditions, and associated with cognitive impairment, reduced quality of life, and increased mortality, partly due to suicide [2–5]. In addition to substantial personal burden, affective disorders impose major societal and economic costs driven by early onset and often recurrent or chronic courses [6, 7]. The sharp increase in incidence and conversion during adolescence and young adulthood [8–10] makes this developmental period critical for understanding the etiopathogenesis of early affective symptomatology. Affective symptoms are dimensionally distributed and show substantial cross-diagnostic overlap, motivating transdiagnostic frameworks that emphasize symptom dimensions [11–14].

Functional magnetic resonance imaging (fMRI) provides a non-invasive method for examining intrinsic large-scale brain organization associated with affective symptoms. Resting-state functional connectivity (RSFC) quantifies temporal correlations in blood-oxygen-level-dependent (BOLD) signals between different brain regions [15] during resting-state fMRI when participants are not engaged in any task [16]. While early RSFC studies assumed connectivity to be stable and thus averaging connectivity across the scan, more recent work conceptualizes RSFC as dynamic, capturing time-varying and transient connectivity states [17].

Key findings of within-network RSFC in depression include changes in frontoparietal (FPN), default mode (DMN), and dorsal attention networks (DAN): Hypoconnectivity within FPN may indicate impairments in cognitive control linked to depression [18], and hypoconnectivity within DAN observed in depressed adolescents [19] may indicate diminished capacity for sustained attention. Hyperconnectivity within DMN has been observed in depressed adolescents and adults [18, 20, 21], potentially reflecting enhanced rumination and negative self-referential thinking. Regarding between-network RSFC, alterations between FPN and DMN have been observed in depression, with most studies reporting increased [18, 22], but some decreased connectivity [21], possibly reflecting difficulties in goal-directed behavior such as shifting attention from internal to external processes. Furthermore, hypoconnectivity between FPN and limbic network (LIM) has been associated with depression [22, 23], specifically between the dorsolateral prefrontal cortex (DLPFC) and the amygdala [24, 25], potentially indicating impairments in top-down emotional regulation. Between DMN and LIM, findings in depression are mixed, with some studies reporting hyper- [22, 25, 26] and others hypoconnectivity [18, 25]. Additionally, reduced connectivity between FPN and DAN has been linked to depression [18], which may reflect compromised cognitive flexibility. Abnormal RSFC of the somatomotor network (SMN) with other networks has also been observed in depression, which could reflect psychomotor inhibition [22, 27]. Dynamic RSFC research indicates that depressed individuals spend more time in connectivity states with overall reduced RSFC and show less frequent transitions between different states compared to healthy controls [28].

Given heterogeneity and low reproducibility of prior findings, likely caused by small samples (typically < 60 participants per group) and methodological differences, further research with larger samples is needed [29, 30]. This study aims to investigate whole-brain static and dynamic RSFC patterns associated with depressive and manic symptom severity in a comparatively large community-based sample of 512 adolescents and young adults, thereby helping to identify candidate vulnerable networks in early affective symptomatology. Such network-level characterization may ultimately inform mechanistic models and guide hypothesis-driven tests of prognostic or intervention relevance in longitudinal work.

Regarding within-network connectivity, we hypothesize that higher depressive symptom severity is associated with decreased RSFC within FPN and DAN, and increased RSFC within DMN, reflecting stronger rumination alongside reduced cognitive and attentional control during the resting-state in depression. Concerning between-network connectivity, we hypothesize that higher depressive symptom severity is associated with decreased connectivity between LIM and FPN and increased connectivity between DMN and FPN, likely reflecting impaired emotional regulation and goal-directed processes in depression. These hypotheses build on most frequently reported findings of altered static RSFC in adolescents and adults with depressive symptoms [18–20, 22, 25]. Given the community-based sample, in contrast to clinical samples in the referenced studies, and heterogeneity of prior findings, we expect tendencies rather than substantial changes with respect to the stated hypotheses. Associations between RSFC and manic symptom severity - as manic symptoms are rare in population-based samples - as well as dynamic RSFC changes, are examined exploratorily.

## Subjects and methods

The study protocol and analysis strategy were preregistered on OSF after participant recruitment but prior to data analysis: https://osf.io/vfzy3/.

### Participants

The present MRI study is part of the Behavior and Mind Health (BeMIND) study [31], a large-scale epidemiological cohort study examining two independent samples of German-speaking age- and sex-stratified adolescents and young adults randomly drawn from the population registry of the city of Dresden, Germany, in 2015 and 2017. All participants of the two BeMIND cohorts were invited to take part in an add-on MRI session. The data collection period of the MRI study spanned from 2016–2018. General exclusion criteria for the BeMIND study comprised insufficient German language skills, institutionalization, or not living in the sampling frame during the baseline assessment period. Additional reasons for exclusion for the add-on MRI-assessment were common MRI-related exclusion criteria (e.g. metal or electronic implants, brackets, epilepsy, claustrophobia, pregnancy), and lack of the MRI-related informed consent (including consent from both legal guardians in minors). Of the 1649 BeMIND baseline participants, 529 participants took part in the MRI session (aged 14–23). Of those, 525 participants completed the structural and resting-state scans, and after quality assurance, data from 512 participants remained.

The description of the MRI data acquisition and preprocessing can be found in the Supplement.

### Affective symptom severity measurement

Current affective symptom severity was assessed by an updated and extended version of the stem-screening-questionnaire [32] which the participants filled out on a tablet after the MRI session. Items of the DSM-5 Self-Rated Level 1 Cross-Cutting Symptom Measure - Adult PROMIS-LEVEL 1 Rating Scale [33] were added. Items were rated by moving a horizontal slider to a position on a five-point scale (0 = none or not at all; 1 = slight or rare, less than a day or two; 2 = mild or several days; 3 = moderate or more than half the days; and 4 = severe or nearly every day) with a 100-point scale underneath (0–19 = category 0/none; 20–39 = category 1/slight; 40–59 = category 2/mild; 60–79 = category 3/moderate; 80–100 = category 4/severe). Depressive and manic symptoms were each assessed by two questions. The mean of these two questions was calculated to create a single measure of depressive and manic symptom severity. Depressive symptoms were measured with these questions: “Have you felt sad or depressed during the past 4 weeks?” and “Have you suffered from loss of interest, tiredness, or lack of energy during the past 4 weeks?”. Manic symptoms were assessed with these questions: “Have you been unusually happy, excited, or irritable during the past 4 weeks, causing friends or family to worry?” and “Have you had an unusual amount of energy during the past 4 weeks, or started more projects than usual, or done more risky things than usual?”.

### Group independent component analysis (GICA)

To evaluate RSFC in the preprocessed resting-state fMRI data, GICA as implemented in the Group ICA of fMRI Toolbox (GIFT) (GroupICATv3.0c; https://trendscenter.org/software/gift/), was used. In spatial GICA [34, 35], as implemented in GIFT, spatially independent sources of BOLD responses are extracted from the resting-state fMRI data. Preprocessing for ICA used variance normalization (z-scoring) and removal of the first five dummy scans. No global signal, tissue-based nuisance, or expanded motion-parameter regression was applied. Data reduction was performed using a two-stage principal component analysis (PCA). In the first stage, each subject’s temporal data were reduced to 120 principal components via a subject-specific PCA. In the second stage, the subject-reduced data were concatenated and further reduced to 100 principal components using a group-wise PCA. To normalize variance, time courses were z-scored as part of the preprocessing in GIFT. ICA was then executed 20 times with the Infomax algorithm [36] in conjunction with ICASSO [37] to evaluate the stability of the extracted components. For each independent component, subject-specific time courses and spatial maps were estimated using spatial-temporal back reconstruction [38]. Subject-specific time courses were detrended, despiked, and filtered using a low-pass filter with high-frequency cut-off of 0.15 Hz. The resulting 100 components were inspected by two independent researchers (P.M.H. and E.M.) and labelled according to anatomical region based on the Automated Anatomical Labelling (AAL) atlas [39] or whether they represented noise, i.e., peak activation outside of grey matter like in ventricles, white matter, blood vessels, and non-brain structures was considered noise. Cerebellar components were excluded due to limited cerebellar head coverage. Furthermore, the spectra of the time courses of meaningful components had to show exponentially higher power at lower frequencies and lower power at higher frequencies. A component is called intrinsic connectivity network (ICN). 53 meaningful ICNs were extracted and subsequently grouped into neurocognitive networks according to the Yeo 7-network solution [40]: visual (VIS), SMN, DAN, ventral attention (VAN), FPN, LIM, and DMN. Additionally, some of the identified ICNs were assigned to a basal ganglia (BG) network. The grouping was achieved by visual inspection and checking the location of the ICN peak coordinates on the 7-network liberal mask in nonlinear MNI152 space, downloaded from: https://surfer.nmr.mgh.harvard.edu/fswiki/CorticalParcellation_Yeo2011 [40].

### Static functional network connectivity (sFNC) analysis

To analyze the association of affective symptom severity and sFNC, a multivariate analysis of covariance (MANCOVA) was performed. As no data on affective symptom severity were available from one participant, *N* = 511 participants were included in this and the following analyses. The sFNC analysis was conducted using the MANCOVAN toolbox implemented in GIFT [41]. The following covariates were included: sex, age, depressive and manic symptom severity, and all two-way interactions of these variables. Motion parameters were used as nuisance regressors. The analysis encompassed multivariate tests followed by univariate tests including only the significant covariates determined in the multivariate stage. An alpha level of 0.01 was employed for all analyses. To correct for multiple comparisons, false discovery rate (FDR) correction [42] was used (*p* < 0.05).

### Dynamic functional network connectivity (dFNC) analysis

The dFNC analysis was performed using the temporal dFNC toolbox implemented in GIFT, which used the time courses of the 53 ICNs obtained from the previous GICA to compute windowed correlation matrices and estimate stable connectivity states. A sliding time-window with a size of 30 TRs (60 s) was convolved with a Gaussian (alpha parameter 3 TRs) to obtain tapering, and for each window an FNC matrix consisting of component pairwise Pearson’s correlations was computed [43]. Identical to the sFNC analysis, variability from motion parameters was regressed out.

To assess the reoccurrence of connectivity patterns over time and across participants, k-means clustering using L1 distance was applied [44]. Based on the elbow criterion, which maximizes within-cluster similarity and between-cluster dissimilarity concurrently, the optimal number of clusters was estimated to be four. Each windowed FNC matrix was assigned to the cluster with which it was most highly correlated. Each dynamic state for each participant is represented by the element-wise median connectivity over all windows assigned to that specific state.

### Model selection

To identify the relevant covariates and to prevent the model from overfitting, a multivariate backward model selection approach that was adapted from the MANCOVAN toolbox implemented in GIFT was used [41]. Statistical models were created for each dynamic state independently, as the covariates may have varying effects on each state. The same covariates as for the sFNC analysis were part of the initial full model for each state.

The following covariates were included in the reduced models of the four states:

- State 1: Sex, age;
- State 2: Sex, depressive symptom severity, manic symptom severity, sex × depressive symptom severity, depressive symptom severity × manic symptom severity;
- State 3: Sex, depressive symptom severity;
- State 4: No significant covariates were identified in the multivariate test.
- Subsequent univariate tests were conducted using these reduced models. Results were FDR-corrected (*p* < 0.05).

### Dynamic indices: Mean dwell time (MDT) and fraction of time (FT)

Two dynamic metrics per state were calculated: MDT, reflecting the average time a participant spent in a particular state before transitioning to another state, and FT (also referred to as state prevalence), indicating the overall proportion of time a participant spent in a particular state [45, 46]. The same backward model selection approach as for the dFNC analysis was used to identify the relevant covariates. The reduced model for MDT included sex, depressive and manic symptom severity, sex × manic symptom severity, and depressive × manic symptom severity. For FT, the reduced model included sex, age, depressive and manic symptom severity, sex × manic symptom severity, age × depressive symptom severity, and depressive × manic symptom severity. FDR-correction (*p* < 0.05) for multiple comparisons was applied on the results.

## Results

### Sample characteristics

Table 1 shows the demographics and affective symptom severity of the participants. Mean age was 18.0 ± 0.1 years. Mean depressive symptom severity was 29.0 ± 0.8, mean manic symptom severity was 14.7 ± 0.6 (calculated on a scale of 0–100). Supplementary Table S1 shows the affective symptom severity separately for the three different age groups. Compared to the total BeMIND sample, participation in the fMRI assessment was more likely for those with older age, high education and those attending university, and less likely for those with non-German nationality.

**Table 1:** Sample characteristics.

	*N*	%
Age		
14–16 years	158	30.9
17–19 years	202	39.4
20–23 years	152	29.7
Sex		
Female	294	57.4
Male	218	42.6
German Nationality		
Yes	506	98.8
No	6	1.2
Living arrangement		
With parents	358	69.9
Alone	53	10.3
With partner	28	5.5
Other	73	14.3
Education		
Low/middle/other	98	19.1
High	414	80.9
Employment		
School	298	58.2
University	117	22.8
Job training	41	8.0
Employed	25	4.9
Unemployed/other	31	6.1
Social class		
Low	66	12.9
Middle	294	57.4
Upper	128	25.0
No information	24	4.7
Financial situation		
Bad	28	5.5
Average	176	34.4
Good	222	43.4
Very good	77	15.0
No information	9	1.7
Marital status		
Married/separated/divorced/widowed	6	1.2
Never married	506	98.8
Depressive symptom severity		
None or not at all	184	35.9
Slight or rare, less than a day or two	207	40.4
Mild or several days	92	18.0
Moderate or more than half the days	24	4.7
Severe or nearly every day	4	0.8
No information	1	0.2
Manic symptom severity		
None or not at all	380	74.2
Slight or rare, less than a day or two	96	18.8
Mild or several days	33	6.4
Moderate or more than half the days	2	0.4
Severe or nearly every day	0	0.0
No information	1	0.2

Demographic characteristics and affective symptom severity of the sample.

### Static FNC

For sFNC, multivariate tests showed effects of sex, age, depressive symptom severity, and an interaction of age and depressive symptom severity. Contrary to our hypotheses, univariate tests of each ICN-to-ICN connection yielded no significant effects of affective symptom severity.

Significant univariate associations with sFNC were only observed for sex and age. Sex effects included both increased and decreased connectivity across all networks (see supplementary Fig. S1), with network-averaged connectivity within the DMN being significantly stronger in females than males. Age showed significant negative associations with sFNC in six ICN-to-ICN connections, including VIS-DAN, VIS-FPN, VIS-DMN, and FPN-DMN connectivity (see supplementary Fig. S2).

### Dynamic FNC

**Fig. 1:**
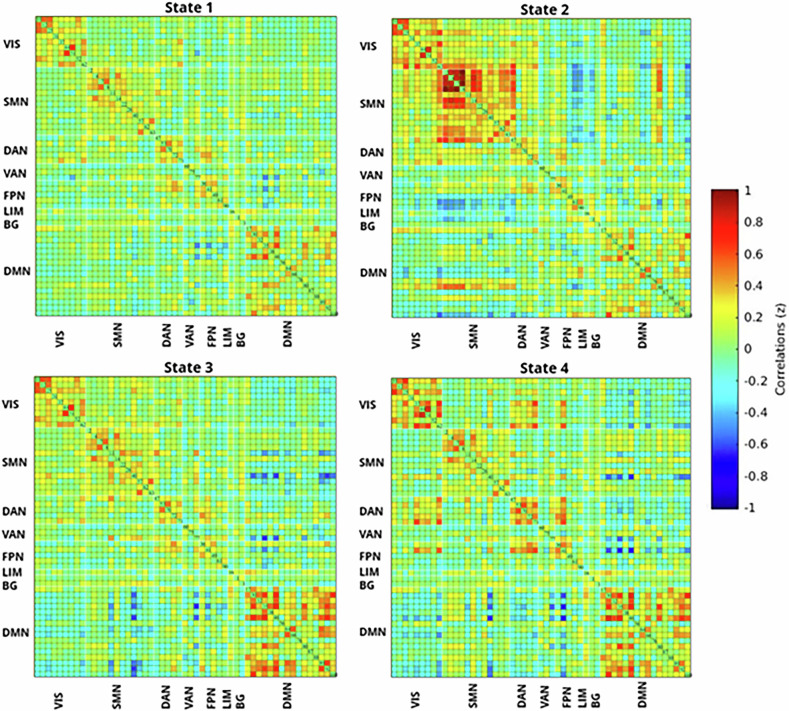
Connectivity matrices of the 4 identified dynamic states. Red indicates positive pairwise connectivity, whereas blue indicates negative pairwise connectivity between the respective components of the 8 neurocognitive networks: visual (VIS), somatomotor (SMN), dorsal attention (DAN), ventral attention (VAN), frontoparietal (FPN), limbic (LIM), basal ganglia (BG), and default mode network (DMN).

**Fig. 2:**
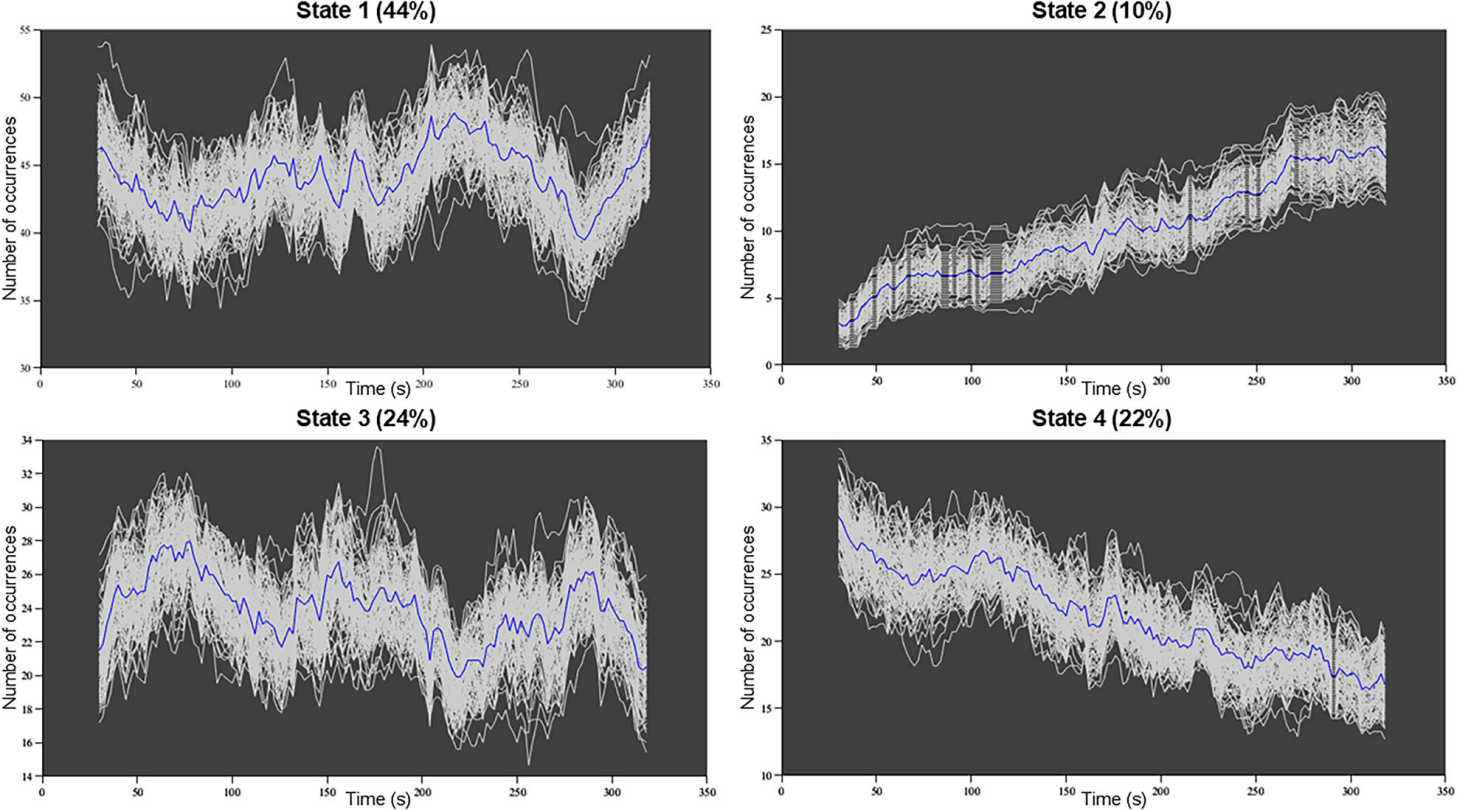
State occurrences over time. The number of occurrences (100 bootstraps) of each state, referring to the percentage of windowed FNC matrices across all participants that are assigned to that state, throughout the scan. Overall occurrence rate was 44% for state 1, 10% for state 2, 24% for state 3, and 22% for state 4.

Connectivity matrices of the resulting 4 dynamic states are shown in Fig. 1. Number of occurrences of each state throughout the scan are depicted in Fig. 2.

State 1 was characterized by showing neither strong positive nor negative connectivity within and between networks. Number of occurrences were relatively steady throughout the duration of the scan. In state 1, 22 ICN-to-ICN connectivity pairs showed significant sex effects (see supplementary Table S4) and 7 connectivity pairs showed significant age effects (see supplementary Table S5).

State 2 was characterized by strong within-VIS and within-SMN connectivity. Negative connectivity was shown between SMN-FPN and SMN-BG. Occurrence rate increased throughout the scan. In state 2, one connectivity pair between SMN and DMN showed a significant effect of sex (see supplementary Table S6). Another connectivity pair between SMN and DMN showed a significant effect of manic symptom severity and of its interaction with depressive symptom severity, respectively (Table 2). A connectivity pair between VIS and SMN showed a significant interaction of sex and depressive symptom severity (Table 2).

**Table 2:** ICN-to-ICN connectivity pairs that show significant effects associated with affective symptom severity in state 2.

ICN 1	ICN 2	Networks	*p*-value	t-value
Significant effect of manic symptom severity				
Postcentral gyrus R + L	Superior temporal gyrus R	SMN-DMN	< 0.001	4.37
Significant effect of the interaction of depressive and manic symptom severity				
Postcentral gyrus R + L	Superior temporal gyrus R	SMN-DMN	< 0.001	−4.41
Significant effect of the interaction of sex and depressive symptom severity				
Middle temporal gyrus R + L	Precuneus R + L	VIS-SMN	< 0.001	−4.36

*ICN* intrinsic connectivity network, *R* right, *L* left, *SMN* somatomotor network, *DMN* default mode network, *VIS* visual network.

State 3 was characterized by positive connectivity within VIS, SMN, and DMN. Negative connectivity was shown between parts of SMN-DMN and VAN-DMN. Number of occurrences were relatively steady throughout the scan. In state 3, 16 ICN-to-ICN connectivity pairs showed significant effects of sex (see supplementary Table S7).

State 4 was characterized by a similar connectivity pattern like state 3, but with stronger within-DAN, between VIS-DAN and DAN-FPN connectivity. State 4 also showed stronger negative connectivity between parts of FPN and DMN compared to state 3. Number of occurrences decreased throughout the scan.

### Dynamic indices: MDT and FT

**Fig. 3:**
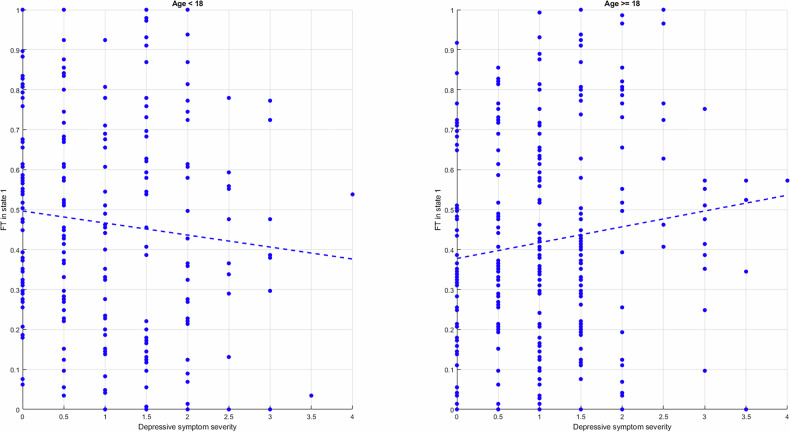
Interaction of age and depressive symptom severity on FT in state 1. For younger age (< 18 years) FT in state 1 decreases with rising depressive symptom severity, for older age (≥ 18 years) FT in state 1 increases with rising depressive symptom severity.

**Fig. 4:**
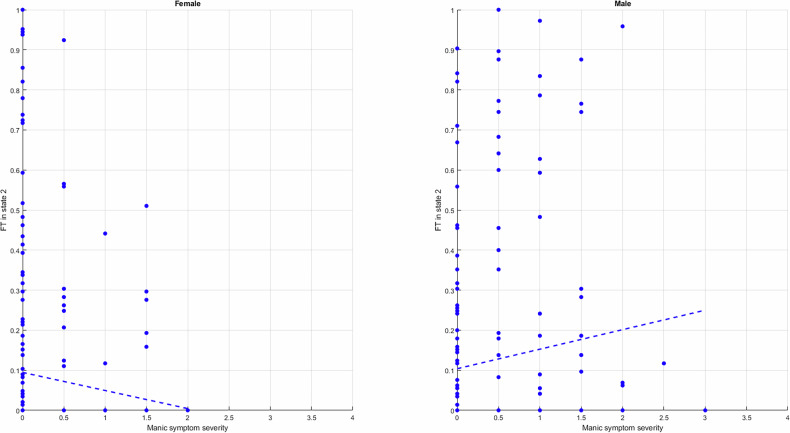
Interaction of sex and manic symptom severity on FT in state 2. For female participants FT in state 2 decreases with rising manic symptom severity, for male participants FT in state 2 increases with rising manic symptom severity.

In the reduced model, MDT demonstrated no significant effects. FT showed significant effects of age in state 1 (*p* < 0.001) and state 3 (*p* = 0.005), an interaction of age and depressive symptom severity (*p* = 0.005; see Fig. 3) and an effect of sex (*p* = 0.002) in state 1, as well as an interaction of sex and manic symptom severity in state 2 (*p* = 0.002; see Fig. 4) in the reduced model.

## Discussion

### Association of affective symptom severity and RSFC

Contrary to our hypotheses, this study did not identify significant univariate associations between affective symptom severity and sFNC within or between the examined networks, although depressive symptom severity showed an effect at the multivariate level. Concerning dFNC, manic symptom severity showed a positive association and its interaction with depressive symptom severity showed a negative association with connectivity between SMN (bilateral postcentral gyrus) and DMN (right superior temporal gyrus) in state 2, which was characterized by strong within-SMN connectivity. Because this effect was restricted to a single connectivity pair, emerged in the state with the lowest occurrence, and resulted from exploratory analyses of manic symptoms and dFNC, it should be regarded as hypothesis-generating and interpreted cautiously.

Prior work has reported decreased SMN-DMN RSFC in depression, including involvement of the right superior temporal gyrus [22], which has also been implicated in RSFC biomarker patterns for depression [47]. Alterations in the postcentral gyrus have also been linked to resting-state abnormalities in depressed patients with hypomanic symptoms [48]. Additionally, bipolar disorder has been associated with mood-state-dependent changes in the balance of resting-state signal variability between SMN and DMN, tilted relatively toward DMN in depression and toward SMN in mania [49], and altered hippocampal-sensorimotor connectivity dynamics have been reported as a vulnerability marker in offspring of bipolar patients [50]. Together, these findings suggest that SMN-DMN-related network dynamics may vary across affective phenotypes, but future studies are needed to test whether SMN-DMN coupling in specific dynamic configurations is reproducible and clinically meaningful. Overall, consistent with our hypothesis that effects would manifest as tendencies rather than substantial connectivity changes, the present findings suggest that future research may benefit from multivariate and dynamic RSFC approaches to detect subtle and transient mood-related deviations that may not be captured by traditional sFNC analyses.

While previous studies have reported substantial alterations in sFNC and dFNC related to depressive symptoms - primarily within and between DMN, FPN and LIM [18, 20, 21, 23, 25, 26, 28, 51] - for the most part, our study did not replicate these findings. One possible explanation may be that our large, community-based cohort of adolescents and young adults generally exhibited low current symptom severity. Consequently, rather temporary and subclinical mood changes in this group may not be accompanied by substantial changes in RSFC, suggesting that they reflect a common, statistically normative mode of brain function in youths. This finding indicates that typical, transient mood fluctuations do not disrupt RSFC on a large scale. Alternatively, RSFC itself may not capture meaningful correlates of subclinical affective variation. In contrast, clinically significant mood disturbances might indeed be associated with marked RSFC changes, as observed in clinical samples [18, 20, 24, 25], even though findings were often controversial and the power of previous studies was significantly lower due to much smaller sample sizes compared to our study. Additional factors, including the heterogeneity of affective symptoms in a community-based sample [52], developmental brain changes during adolescence [53, 54], and the increased variability introduced by dFNC [17], may further reduce the likelihood of detecting significant group-level RSFC alterations.

Given that age and sex effects were not the primary focus of this study, they are discussed in the Supplement.

### Interpretation of dynamic connectivity states

The dFNC analysis revealed four reoccurring states characterized by varying connectivity patterns. State 1 showed comparatively weak connectivity across network pairs, a pattern consistent with reports of a frequently occurring, low-coupling configuration in resting-state dFNC studies [55]. Given that age and sex effects were observed and that FT in state 1 was significantly influenced by age and sex, this baseline connectivity mode may be modulated by demographic factors [56]. The interaction between age and depressive symptom severity on FT in state 1 further indicates that associations between depressive symptoms and brain dynamics may differ across developmental stages. State 2 was characterized by positive connectivity within VIS and SMN and negative connectivity between SMN and FPN, and its occurrence increased across the scan. While similar temporal trends and connectivity patterns have previously been discussed in relation to emerging tiredness over the course of resting-state acquisitions [57], such interpretations remain tentative here in the absence of direct measures. The observed interaction between sex and manic symptom severity on FT in state 2 suggests that symptom-related modulation of these connectivity dynamics may differ by sex. State 3 exhibited enhanced within-DMN connectivity relative to states 1 and 2 that appeared intermittently during the scan. Sex effects and an age effect on FT in state 3 indicate that this connectivity configuration and the likelihood of occupying it varies across demographic groups. State 4 decreased in occurrence over the scan, which in line with prior observations may represent a transient phase of vigilance that diminishes as participants become tired [55], but future studies incorporating physiological measures are needed to determine the functional relevance of these states.

### Limitations

First, the assessment of affective symptoms in the present study was relatively brief, comprising two self-report questions each assessing depressive and manic symptoms. These were chosen because they were the only measures of affective symptoms administered on the same day as the fMRI scan. More extensive instruments were collected during the baseline assessment, but with a substantial and highly variable time lag relative to MRI acquisition ranging from several weeks to several years, rendering them unsuitable for meaningful brain-symptom association analyses. Although ultra-brief measures are inherently limited, prior work indicates that even single-item screening questions can capture clinically relevant affective symptom variance, albeit efficacy varies across populations and settings [58, 59]. To address this limitation, validation analyses against established instruments are provided in the Supplement.

Second, adolescence is a developmental period of substantial neuronal changes and synaptic pruning. Although age was included as a covariate in all analyses, some relevant effects may still be masked, as significant differences in brain maturation are expected across the 14–23-year age range studied and age may nonlinearly moderate symptom-RSFC associations [53, 60]. While subgroup analyses for narrower age ranges would be of interest, such stratification would have substantially reduced statistical power. Given growing evidence that reliable and reproducible RSFC estimates require large samples [29, 30], we prioritized maximizing sample size to improve stability and generalizability of the findings. Future large-scale studies will be better positioned to explicitly investigate age-specific effects.

Third, cerebellar components were excluded from the analyses, limiting conclusions regarding cerebellar contributions to affective symptomatology. In addition, although motion correction and quality control procedures were applied, head motion remains a challenge especially in adolescent fMRI and residual motion-related effects on connectivity estimates cannot be fully excluded [61, 62]. Moreover, because sliding-window dFNC remains debated and reliability depends on analytic choices and data length, these results should be interpreted cautiously and primarily as hypothesis-generating, and future studies should consider alternative analytic approaches [63, 64].

Fourth, additional factors known to influence RSFC, including medication, sleep patterns, comorbid psychopathology, and handedness were not explicitly modeled in the present analyses. Given the dimensional, population-based focus of the study, these factors likely contribute to the natural heterogeneity of affective symptom expressions in the community. Accordingly, the reported associations should be interpreted at the population level. In addition, caffeine intake and sleep prior to scanning were not standardized, and scan-time vigilance was not monitored, which may have introduced additional variability in RSFC estimates.

Fifth, as the study protocol and analysis strategy were developed several years after participant recruitment and data acquisition, preregistration necessarily occurred post hoc, limiting the extent to which preregistration can rule out data-dependent analytic decisions.

Finally, although all BeMIND participants were invited to the fMRI study, participation was selective with respect to demographic characteristics, which may limit the representativeness of the fMRI subsample.

## Conclusion

In this large community-based sample of over 500 adolescents and young adults, we did not identify significant univariate associations between affective symptom severity and static RSFC. Dynamic RSFC analysis revealed a single state-specific association between manic symptom severity and SMN-DMN connectivity, as well as interactions of affective symptoms with age and sex in the dynamic index FT. Overall, these findings suggest that RSFC measures show limited sensitivity to subclinical affective symptom variation in community youth and underscore the challenges of identifying reliable RSFC markers of affective symptoms, especially outside clinical populations. Future research will be required to determine whether more refined analytic approaches, longitudinal designs, or clinical samples are necessary to detect meaningful RSFC-symptom associations.

## Supplementary information

Supplement

## Data Availability

The data supporting the findings of this study are not publicly available due to ethical and data protection restrictions concerning the privacy of human research participants. The minimum dataset required to interpret and verify the findings may be made available upon reasonable request to the corresponding author, P.M.H. (PaulaMarie.Henneberg@ukdd.de). Requests will be reviewed in accordance with the consent provided by participants and applicable institutional and legal requirements.
