# Supplementary material for: Changes in resting-state functional connectivity linked to affective symptoms: insights from a population-based study of adolescents and young adults
Source: Transl Psychiatry. 2026 Jul 15;16:362. doi: 10.1038/s41398-026-04269-y (PMC13369973; doi:10.1038/s41398-026-04269-y)
Supplement: Supplementary file 1 — Supplement [file 41398_2026_4269_MOESM1_ESM.docx]

Supplement

Methods

MRI data acquisition

The MRI assessment consisted of a high-resolution structural scan with a length of seven minutes, directly followed by a resting-state scan with a length of six minutes, after which two fMRI-tasks were completed in the scanner. All MRI data were obtained using a 3-Tesla Siemens Tim Trio scanner with the Siemens 32-channel head coil (Siemens). T1-weighted images were acquired with a 3D magnetization-prepared rapid gradient echo (MPRAGE) sequence (repetition time [TR] = 1.9 s, echo time [TE] = 2.26 ms, field of view [FOV] = 256 mm x 224 mm x 176 mm, voxel size = 1 mm x 1 mm x 1 mm, inversion time = 0.9 s, flip angle [FA] = 9°, phase partial Fourier 7/8, bandwidth [BW] = 200 Hz/Px). Functional resting-state data (180 volumes) were acquired with an EPI sequence (TR = 2 s, TE = 25.0 ms, FOV = 192 mm x 192 mm x 132 mm, voxel size = 3.2 mm x 3.2 mm x 4 mm, slice thickness of 3.2 mm with 25 % slice gap, 34 slices, descending order, tilted approximately -15° from axial to coronal, flip angle = 78°, weak raw data low-pass filter, band width = 2004 Hz/Px). Participants were instructed to stay awake, fixate a crosshair on a display and allow their minds to wander during the scan.

MRI data preprocessing

The resting-state fMRI data were preprocessed using SPM12 standalone version 7771 (Wellcome Centre for Human Neuroimaging) with MATLAB version R2019b (MathWorks). Functional images were corrected for slice timing and gross head motion. Next, the mean image was coregistered to each participant's T1-weighted MR image using the normalized mutual information algorithm. T1-weighted images were then normalized to the “ICBM space template - European Brains” that is part of SPM12. Using the transform parameters of the normalization of the T1-weighted image, functional images were normalized to MNI space and resampled into 3 mm isotropic voxels. Finally, normalized images were spatially smoothed with a Gaussian kernel with a FWHM of 6 x 6 x 6 mm.

The fMRI data quality assurance procedure included examination of the motion parameters and visual inspection of the EPI video for fMRI artefacts, coregistration, and normalization. Head motion was summarized as the fraction of volumes exceeding framewise displacements > 0.5 mm: Across the total sample of 525 participants, this mean fraction was 2.14 ± 5.32. The fraction of framewise displacements exceeding 0.5 mm was not meaningfully associated with depressive or manic symptom severity (all p > 0.05). Data from 13 participants were excluded due to excessive head motion - defined as more than 7.5 % of framewise displacements exceeding 0.8 mm (1) - or due to MR image artifacts, resulting in a sample of 512 participants included in subsequent analyses.

Results: Sample characteristics

Table S1. Affective symptom severity in the three different age groups.

|  | Age (years) | | |  |
| --- | --- | --- | --- | --- |
|  | 14-16 | 17-19 | 20-23 | Total |
| Depressive symptom severity |  |  |  |  |
| None or not at all | 74 | 61 | 49 | 184 |
| Row % | 40.2 | 33.2 | 26.6 | 100.0 |
| Column % | 47.1 | 30.2 | 32.2 | 36.0 |
| Slight or rare, less than a day or two | 46 | 91 | 70 | 207 |
| Row % | 22.2 | 44.0 | 33.8 | 100.0 |
| Column % | 29.3 | 45.1 | 46.0 | 40.5 |
| Mild or several days | 31 | 34 | 27 | 92 |
| Row % | 33.7 | 37.0 | 29.3 | 100.0 |
| Column % | 19.8 | 16.8 | 17.8 | 18.0 |
| Moderate or more than half the days | 5 | 16 | 3 | 24 |
| Row % | 20.8 | 66.7 | 12.5 | 100.0 |
| Column % | 3.2 | 7.9 | 2.0 | 4.7 |
| Severe or nearly every day | 1 | 0 | 3 | 4 |
| Row % | 25.0 | 0.0 | 75.0 | 100.0 |
| Column % | 0.6 | 0.0 | 2.0 | 0.8 |
| Total | 157 | 202 | 152 | 511 |
| Row % | 30.7 | 39.5 | 29.8 | 100.0 |
| Column % | 100.0 | 100.0 | 100.0 | 100.0 |
| Manic symptom severity |  |  |  |  |
| None or not at all | 114 | 147 | 119 | 380 |
| Row % | 30.0 | 38.7 | 31.3 | 100.0 |
| Column % | 72.6 | 72.8 | 78.3 | 74.3 |
| Slight or rare, less than a day or two | 31 | 36 | 29 | 96 |
| Row % | 32.3 | 37.5 | 30.2 | 100.0 |
| Column % | 19.7 | 17.8 | 19.1 | 18.8 |
| Mild or several days | 10 | 19 | 4 | 33 |
| Row % | 30.3 | 57.6 | 12.1 | 100.0 |
| Column % | 6.4 | 9.4 | 2.6 | 6.5 |
| Moderate or more than half the days | 2 | 0 | 0 | 2 |
| Row % | 100.0 | 0.0 | 0.0 | 100.0 |
| Column % | 1.3 | 0.0 | 0.0 | 0.4 |
| Severe or nearly every day | 0 | 0 | 0 | 0 |
| Row % | 0.0 | 0.0 | 0.0 | 0.0 |
| Column % | 0.0 | 0.0 | 0.0 | 0.0 |
| Total | 157 | 202 | 152 | 511 |
| Row % | 30.7 | 39.5 | 29.8 | 100.0 |
| Column % | 100.0 | 100.0 | 100.0 | 100.0 |

Distribution of depressive and manic symptom severity in the three age groups.

Results: Static FNC

Supplementary Figure S1. Connectogram of ICNs showing sex effects in sFNC.


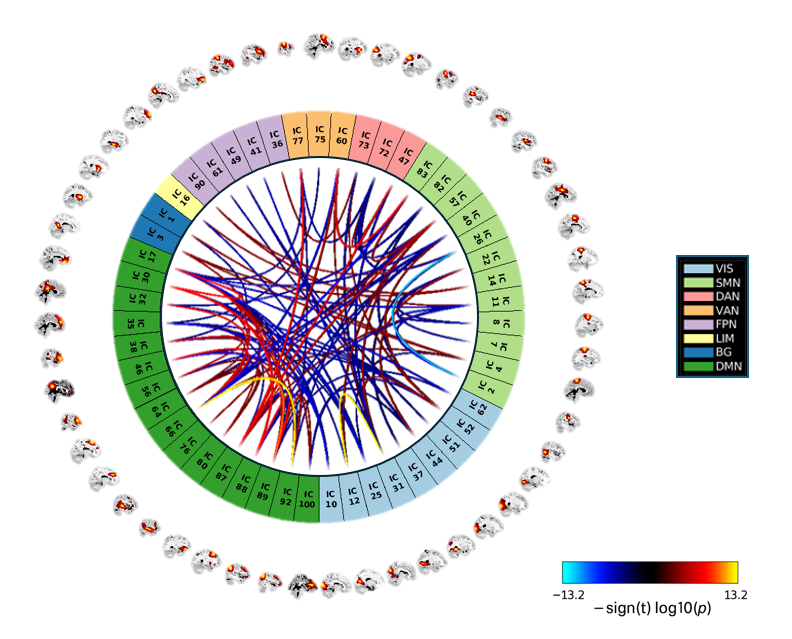


Significant differences (p < 0.01) in sFNC between females compared to males in the visual (VIS), somatomotor (SMN), dorsal attention (DAN), ventral attention (VAN), frontoparietal (FPN), limbic (LIM), basal ganglia (BG), and default mode network (DMN). Yellow and red connections between two independent components (IC) indicate stronger connectivity in females, blue connections indicate stronger connectivity in males.

Table S2. ICN-to-ICN connectivity pairs that show strongest significant sex effects in sFNC.

| ICN 1 | ICN 2 | Networks | p-value | t-value | Mean connectivity | | Relationship |
| --- | --- | --- | --- | --- | --- | --- | --- |
|  |  |  |  |  | Male | Female |  |
| Calcarine sulcus R+L (12) | Lingual gyrus R+L (31) | VIS-VIS | < 0.001 | 7.71 | 0.21 | 0.34 | male < female |
| Superior frontal gyrus (SFG) R+L (66) | Inferior parietal lobule L (92) | DMN-DMN | < 0.001 | 7.70 | 0.26 | 0.39 | male < female |
| Postcentral gyrus R+L (2) | Midcingulate cortex (MCC) R+L (26) | SMN-SMN | < 0.001 | -6.83 | 0.17 | 0.05 | male > female |
| Superior frontal gyrus (SFG) R+L (66) | Inferior parietal lobule R (89) | DMN-DMN | < 0.001 | 6.43 | 0.30 | 0.42 | male < female |
| Superior frontal gyrus (SFG) R+L (88) | Inferior parietal lobule R (89) | DMN-DMN | < 0.001 | 5.72 | 0.07 | 0.17 | male < female |
| Rostral anterior cingulate cortex (rACC) R+L (30) | Superior frontal gyrus (SFG) R+L (88) | DMN-DMN | < 0.001 | 5.63 | 0.25 | 0.35 | male < female |
| Superior temporal gyrus (STG) R+L (83) | Anterior insula R+L (60) | SMN-VAN | < 0.001 | 5.57 | 0.03 | 0.13 | male < female |
| Superior temporal gyrus (STG) R+L (83) | Superior frontal gyrus (SFG) R+L (66) | SMN-DMN | < 0.001 | -5.56 | -0.16 | -0.24 | male > female |
| Supramarginal gyrus R+L (77) | Orbitofrontal cortex (OFC) R+L (87) | VAN-DMN | < 0.001 | 5.50 | 0.03 | 0.11 | male < female |
| Precuneus R+L (17) | Rostral anterior cingulate cortex (rACC) R+L (30) | DMN-DMN | < 0.001 | 5.46 | 0.32 | 0.42 | male < female |

R: right; L: left; VIS: visual network; DMN: default mode network; SMN: somatomotor network; VAN: ventral attention network.

Supplementary Figure S2. Connectogram of ICNs showing age effects in sFNC.


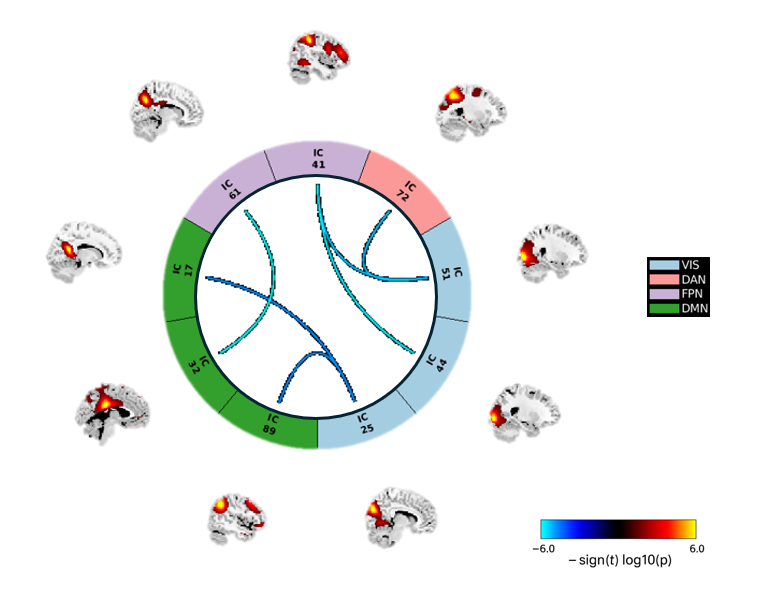


Significant differences (p < 0.01) in sFNC in the visual (VIS), dorsal attention (DAN), frontoparietal (FPN), and default mode network (DMN) related to age. Blue connections between two independent components (IC) indicate weaker connectivity in older compared to younger age.

Table S3. ICN-to-ICN connectivity pairs that show significant age effects in sFNC.

| ICN 1 | ICN 2 | Networks | p-value | t-value |
| --- | --- | --- | --- | --- |
| cuneus R+L (25) | precuneus R+L (17) | VIS-DMN | < 0.001 | -4.26 |
| cuneus R+L (25) | inferior parietal lobule R (89) | VIS-DMN | < 0.001 | -4.26 |
| cuneus L (44) | intraparietal sulcus (IPS) R (41) | VIS-FPN | < 0.001 | -4.62 |
| middle occipital gyrus (MOG) R (51) | superior parietal lobule R+L (72) | VIS-DAN | < 0.001 | -4.41 |
| middle occipital gyrus (MOG) R (51) | intraparietal sulcus (IPS) R (41) | VIS-FPN | < 0.001 | -4.47 |
| precuneus R+L (61) | posterior cingulate cortex (PCC) R+L (32) | FPN-DMN | < 0.001 | -4.94 |

FPN: frontoparietal network; DAN: dorsal attention network; other abbreviations are explained in the footnote of Table S2.

Results: Dynamic FNC

Table S4. ICN-to-ICN connectivity pairs that show significant effects of sex in state 1.

| ICN 1 | ICN 2 | Networks | p-value | t-value | Mean connectivity | | Relationship |
| --- | --- | --- | --- | --- | --- | --- | --- |
|  |  |  |  |  | Male | Female |  |
| Calcarine sulcus R+L | Lingual gyrus R+L | VIS-VIS | < 0.001 | 4.71 | 0.20 | 0.31 | male < female |
| Cuneus L | Middle frontal gyrus R+L | VIS-FPN | < 0.001 | -3.59 | -0.06 | -0.15 | male > female |
| Middle temporal gyrus R+L | Putamen R+L | VIS-BG | < 0.001 | 4.09 | 0.02 | 0.10 | male < female |
| Postcentral gyrus R+L | Primary motor cortex R+L | SMN-SMN | < 0.001 | -3.54 | 0.38 | 0.27 | male > female |
| Postcentral gyrus R+L | Midcingulate cortex R+L | SMN-SMN | < 0.001 | -4.42 | 0.12 | 0.01 | male > female |
| Primary motor cortex R | Premotor cortex R+L | SMN-DAN | < 0.001 | -3.53 | 0.14 | 0.06 | male > female |
| Precuneus R+L | Orbitofrontal cortex R+L | SMN-DMN | < 0.001 | 4.02 | -0.11 | -0.02 | male < female |
| Precuneus R+L | Superior frontal gyrus R+L | SMN-DMN | < 0.001 | -4.71 | 0.14 | 0.04 | male > female |
| Primary motor cortex R+L | Hippocampus/amygdala R+L | SMN-LIM | < 0.001 | 3.56 | 0.03 | 0.10 | male < female |
| Midcingulate cortex R+L | Superior temporal gyrus R+L | SMN-SMN | < 0.001 | -3.75 | 0.12 | 0.04 | male > female |
| Primary motor cortex R+L | Superior frontal gyrus R+L | SMN-DMN | < 0.001 | -3.66 | -0.07 | -0.16 | male > female |
| Posterior insula R+L | Orbitofrontal cortex R+L | SMN-DMN | < 0.001 | 4.18 | 0.00 | 0.10 | male < female |
| Superior temporal gyrus R+L | Posterior cingulate cortex R+L | SMN-DMN | < 0.001 | -4.45 | 0.13 | 0.03 | male > female |
| Superior temporal gyrus R+L | Superior parietal lobule R+L | SMN-DAN | < 0.001 | -3.60 | -0.19 | -0.26 | male > female |
| Superior temporal gyrus R+L | Superior frontal gyrus R+L | SMN-DMN | < 0.001 | -5.29 | -0.14 | -0.24 | male > female |
| Inferior parietal lobule R+L | Superior parietal lobule R+L | DAN-DAN | 0.001 | 3.50 | 0.09 | 0.17 | male < female |
| Inferior parietal lobule R+L | Orbitofrontal cortex R+L | DAN-DMN | < 0.001 | 3.65 | -0.03 | 0.05 | male < female |
| Precuneus R+L | Superior frontal gyrus R+L | DMN-DMN | < 0.001 | 3.61 | -0.04 | 0.04 | male < female |
| Rostral anterior cingulate cortex (rACC) R+L | Inferior frontal gyrus L | DMN-DMN | < 0.001 | -3.77 | 0.05 | -0.05 | male > female |
| Inferior frontal gyrus L | Superior frontal gyrus R+L | DMN-DMN | < 0.001 | 3.52 | 0.07 | 0.15 | male < female |
| Superior frontal gyrus R+L | Inferior parietal lobule R | DMN-DMN | 0.001 | 3.50 | 0.26 | 0.34 | male < female |
| Superior frontal gyrus R+L | Inferior parietal lobule L | DMN-DMN | < 0.001 | 5.65 | 0.19 | 0.32 | male < female |

LIM: limbic network; BG: basal ganglia network; other abbreviations are explained in the footnote of Table S2 and S3.

Table S5. ICN-to-ICN connectivity pairs that show significant effects of age in state 1.

| ICN 1 | ICN 2 | Networks | p-value | t-value |
| --- | --- | --- | --- | --- |
| Primary visual cortex R+L | Inferior parietal lobule R | VIS-DMN | < 0.001 | -3.54 |
| Calcarine sulcus R+L | Rostral anterior cingulate cortex (rACC) R+L | VIS-DMN | < 0.001 | -3.72 |
| Middle occipital gyrus R | Superior parietal lobule R+L | VIS-DAN | < 0.001 | -3.65 |
| Middle occipital gyrus R | Intraparietal sulcus R | VIS-FPN | < 0.001 | -3.61 |
| Precuneus R+L | Posterior cingulate cortex R+L | FPN-DMN | < 0.001 | -3.51 |
| Precuneus R+L | Inferior parietal lobule R | FPN-DMN | < 0.001 | -3.55 |
| Inferior frontal gyrus R | Superior frontal gyrus R+L | DMN-DMN | < 0.001 | -3.74 |

Abbreviations are explained in the footnote of Table S2 and S3. Negative t-values imply a decrease in connectivity with age.

Table S6. ICN-to-ICN connectivity pair that shows a significant effect of sex in state 2.

| ICN 1 | ICN 2 | Networks | p-value | t-value | Mean connectivity | | Relationship |
| --- | --- | --- | --- | --- | --- | --- | --- |
|  |  |  |  |  | Male | Female |  |
| Posterior insula R+L | Superior temporal gyrus R | SMN-DMN | < 0.001 | 4.66 | 0.01 | 0.13 | male < female |

Abbreviations are explained in the footnote of Table S2.

Table S7. ICN-to-ICN connectivity pairs that show significant effects of sex in state 3.

| ICN 1 | ICN 2 | Networks | p-value | t-value | Mean connectivity | | Relationship |
| --- | --- | --- | --- | --- | --- | --- | --- |
|  |  |  |  |  | Male | Female |  |
| Calcarine sulcus R+L | Lingual gyrus R+L | VIS-VIS | < 0.001 | 3.95 | 0.24 | 0.35 | male < female |
| Lingual gyrus R+L | Anterior insula R+L | VIS-VAN | < 0.001 | 4.82 | 0.01 | 0.13 | male < female |
| Lingual gyrus R+L | Superior frontal gyrus R+L | VIS-DMN | < 0.001 | -3.73 | 0.09 | -0.02 | male > female |
| Lingual gyrus R+L | Rostral anterior cingulate cortex (rACC) R+L | VIS-DMN | < 0.001 | -3.82 | -0.10 | -0.20 | male > female |
| Postcentral gyrus R+L | Midcingulate cortex R+L | SMN-SMN | < 0.001 | -3.77 | 0.12 | 0.00 | male > female |
| Postcentral gyrus R+L | Intraparietal sulcus R+L | SMN-DAN | < 0.001 | 3.95 | -0.02 | 0.09 | male < female |
| Posterior insula R+L | Orbitofrontal cortex R+L | SMN-DMN | < 0.001 | 4.50 | -0.13 | 0.01 | male < female |
| Supramarginal gyrus R+L | Orbitofrontal cortex R+L | VAN-DMN | < 0.001 | 5.67 | -0.08 | 0.09 | male < female |
| Rostral anterior cingulate cortex (rACC) R+L | Superior frontal gyrus R+L | DMN-DMN | < 0.001 | 4.18 | 0.29 | 0.40 | male < female |
| Rostral anterior cingulate cortex (rACC) R+L | Superior frontal gyrus R+L | DMN-DMN | < 0.001 | 4.29 | 0.30 | 0.42 | male < female |
| Ventromedial prefrontal cortex (VMPFC) R+L | Orbitofrontal cortex R+L | DMN-DMN | < 0.001 | -3.82 | 0.27 | 0.14 | male > female |
| Precuneus R+L | Superior frontal gyrus R+L | DMN-DMN | < 0.001 | 4.07 | 0.33 | 0.46 | male < female |
| Precuneus R+L | Superior frontal gyrus R+L | DMN-DMN | < 0.001 | 3.74 | 0.29 | 0.40 | male < female |
| Superior frontal gyrus R+L | Inferior parietal lobule L | DMN-DMN | < 0.001 | 5.17 | 0.38 | 0.54 | male < female |
| Orbitofrontal cortex R+L | Inferior parietal lobule R | DMN-DMN | < 0.001 | -4.34 | 0.14 | 0.01 | male > female |
| Superior frontal gyrus R+L | Inferior parietal lobule R | DMN-DMN | < 0.001 | 5.08 | 0.09 | 0.25 | male < female |

Abbreviations are explained in the footnote of Table S2 and S3.

Discussion of age and sex effects

Sex effects

Sex showed both significant positive and negative associations with sFNC in numerous connections including all networks (Figure S1 and Table S2). The dFNC analysis yielded similar results, with significant sex effects in states 1, 2, and 3 including all networks and both significantly increased and decreased intra- and inter-network functional connectivity (Table S4, S6, and S7). Previous studies also reported sex differences in attention, salience, frontoparietal, sensorimotor, and visual networks (2–5), consistent with the results of the present study. However, few previous studies reported that there were no significant differences between sexes in RSFC (6).

Connectivity within the DMN was shown to be significantly stronger in females compared to males in the sFNC analysis which is consistent with prior reports in adolescents and adults (2,3,7,8).

Age effects

In the sFNC analysis, we observed significant age-related decreases in between-network functional connectivity involving VIS-DAN, VIS-FPN, VIS-DMN, and FPN-DMN connectivity pairs (Figure S2 and Table S3). In the dFNC analysis, highly similar age effects were observed in state 1, involving the same networks identified in the sFNC analysis (Table S5). In addition, an age-related decrease in intra-network functional connectivity was detected within the DMN for one connectivity pair in state 1.

These results reflect previous findings in the literature showing that functional connectivity between resting-state networks decreases with age throughout adolescence (8,9). Matching our results, age-related decreased RSFC has been observed particularly between the VIS and other networks (10). Decreasing inter-network connectivity likely reflects increasing segregation between and specialization of functionally distinct brain networks with ongoing adolescent maturation. Additionally, functional connectivity within resting-state networks was shown to increase throughout adolescence (8,9), likely reflecting increasing integration within brain networks and thus improving communication between functionally related regions, which was not observed in our study. The pattern of decreasing between-network functional connectivity and increasing within-network functional connectivity has been reported to be reversed in older age (11). Thus, reduced connectivity within resting-state networks and increased connectivity between resting-state networks has been associated with cognitive decline in older adults, with networks becoming less efficient and differentiated (12).

Validation of symptom assessment

As a validation against established psychometric tools we calculated the correlation of the depressive symptom severity measured on the day of the fMRI scan and the PHQ‐9 (13) as well as the PROMIS (14) measured during the baseline assessment of the BeMIND study. Correlation with both instruments was moderate (0.45) but could be regarded as strong considering the time gap between baseline and fMRI assessment.

Depressive symptom severity measured on the day of the fMRI scan was significantly associated with presence of major depressive episode(s) assessed face-to-face using an updated version of the Munich Composite International Diagnostic Interview (DIA-X-5; 15) during the baseline assessment of the BeMIND study (past 12 months: Odds Ratio (OR) = 2.7, 95%-Confidence Interval (CI): 1.8-3.9, p < 0.001; lifetime: OR = 2.3, CI: 1.7-3.1, p < 0.001).

There was a weak correlation of the manic symptom severity measured on the day of the fMRI scan with the symptoms of mania as assessed by the DSM‐5 Self‐Rated Level 1 Cross‐Cutting Symptom Measure (0.23) and the Altman Self‐Rating Mania Scale (ASRM; 16; correlation 0.15) measured during the baseline assessment of the BeMIND study. It is not surprising that the correlation was weak as there was a time gap between baseline and fMRI assessment that exceeds the typical duration of manic or hypomanic episodes.

Manic symptom severity measured on the day of the fMRI scan was significantly associated with manic or hypomanic episode(s) assessed via the clinical diagnostic interview during the baseline assessment of the BeMIND study (past 12 months: OR = 4.6, 95%-CI: 1.9-10.6, p < 0.001; lifetime: OR = 3.3, CI: 1.7-6.4, p < 0.001).

References

1. Power JD, Barnes KA, Snyder AZ, Schlaggar BL, Petersen SE. Spurious but systematic correlations in functional connectivity MRI networks arise from subject motion. NeuroImage. 2012 Feb;59(3):2142–54. doi:10.1016/j.neuroimage.2011.10.018

2. Allen EA, Erhardt EB, Damaraju E, Gruner W, Segall JM, Silva RF, et al. A baseline for the multivariate comparison of resting-state networks. Front Syst Neurosci. 2011;5:2. doi:10.3389/fnsys.2011.00002 PubMed PMID: 21442040; PubMed Central PMCID: PMC3051178.

3. Biswal BB, Mennes M, Zuo XN, Gohel S, Kelly C, Smith SM, et al. Toward discovery science of human brain function. Proc Natl Acad Sci. 2010 Mar 9;107(10):4734–9. doi:10.1073/pnas.0911855107

4. Filippi M, Valsasina P, Misci P, Falini A, Comi G, Rocca MA. The organization of intrinsic brain activity differs between genders: A resting‐state fMRI study in a large cohort of young healthy subjects. Hum Brain Mapp. 2013 Jun;34(6):1330–43. doi:10.1002/hbm.21514

5. Scheinost D, Finn ES, Tokoglu F, Shen X, Papademetris X, Hampson M, et al. Sex differences in normal age trajectories of functional brain networks. Hum Brain Mapp. 2015 Apr;36(4):1524–35. doi:10.1002/hbm.22720

6. Weissman‐Fogel I, Moayedi M, Taylor KS, Pope G, Davis KD. Cognitive and default‐mode resting state networks: Do male and female brains “rest” differently? Hum Brain Mapp. 2010 Nov;31(11):1713–26. doi:10.1002/hbm.20968

7. Jamadar SD, Sforazzini F, Raniga P, Ferris NJ, Paton B, Bailey MJ, et al. Sexual Dimorphism of Resting-State Network Connectivity in Healthy Ageing. Anderson N, editor. J Gerontol Ser B. 2019 Sep 15;74(7):1121–31. doi:10.1093/geronb/gby004

8. Teeuw J, Brouwer RM, Guimarães JPOFT, Brandner P, Koenis MMG, Swagerman SC, et al. Genetic and environmental influences on functional connectivity within and between canonical cortical resting-state networks throughout adolescent development in boys and girls. NeuroImage. 2019 Nov;202:116073. doi:10.1016/j.neuroimage.2019.116073

9. Sherman LE, Rudie JD, Pfeifer JH, Masten CL, McNealy K, Dapretto M. Development of the Default Mode and Central Executive Networks across early adolescence: A longitudinal study. Dev Cogn Neurosci. 2014 Oct;10:148–59. doi:10.1016/j.dcn.2014.08.002

10. Rosenberg BM, Mennigen E, Monti MM, Kaiser RH. Functional Segregation of Human Brain Networks Across the Lifespan: An Exploratory Analysis of Static and Dynamic Resting-State Functional Connectivity. Front Neurosci. 2020 Dec 8;14:561594. doi:10.3389/fnins.2020.561594

11. Betzel RF, Byrge L, He Y, Goñi J, Zuo XN, Sporns O. Changes in structural and functional connectivity among resting-state networks across the human lifespan. NeuroImage. 2014 Nov;102:345–57. doi:10.1016/j.neuroimage.2014.07.067

12. Geerligs L, Renken RJ, Saliasi E, Maurits NM, Lorist MM. A Brain-Wide Study of Age-Related Changes in Functional Connectivity. Cereb Cortex. 2015 Jul;25(7):1987–99. doi:10.1093/cercor/bhu012

13. Kroenke K, Spitzer RL, Williams JBW. The PHQ-9: Validity of a brief depression severity measure. J Gen Intern Med. 2001 Sep;16(9):606–13. doi:10.1046/j.1525-1497.2001.016009606.x

14. Cella D, Riley W, Stone A, Rothrock N, Reeve B, Yount S, et al. The Patient-Reported Outcomes Measurement Information System (PROMIS) developed and tested its first wave of adult self-reported health outcome item banks: 2005–2008. J Clin Epidemiol. 2010 Nov;63(11):1179–94. doi:10.1016/j.jclinepi.2010.04.011

15. Hoyer J, Voss C, Strehle J, Venz J, Pieper L, Wittchen HU, et al. Test-retest reliability of the computer-assisted DIA-X-5 interview for mental disorders. BMC Psychiatry. 2020 Dec;20(1):280. doi:10.1186/s12888-020-02653-6

16. Altman EG, Hedeker D, Peterson JL, Davis JM. The Altman Self-Rating Mania Scale. Biol Psychiatry. 1997 Nov;42(10):948–55. doi:10.1016/S0006-3223(96)00548-3
